# Keynote 2: Jane ThorntonA call for “movement equity”: What (and who) are we still missing in the conversation on physical activity and health?

**DOI:** 10.1093/eurpub/ckae114.002

**Published:** 2024-09-26

## Abstract

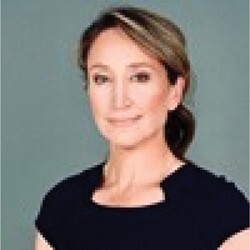

Finding guidance on evidence-based physical activity counselling in healthcare can be challenging, and patients and providers are often unsure about what and how much exercise is needed. This session will introduce some of the work of Dr. Thornton’s lab to facilitate access to physical activity and take the guesswork out of counselling on how to be active.

Working with patients and community partners directly has provided much-needed insight into the barriers and opportunities patients face when approaching physical activity. Dr. Thornton will outline co-produced steps involved in engaging patients in physical activity research and the results of her work at the local community level that have steered the work to focus on more equitable, inclusive and diverse access to movement.

Dr Thornton will introduce the term movement equity and define a 5-step framework to help create movement equity in sport and exercise medicine settings. With equity at the centre of our physical activity counselling strategies, we can encourage a culture a movement across the lifespan and around the world.

